# Current Prevalence of Rubella Antibodies in Pregnant Women at a Japanese Perinatal Center

**DOI:** 10.31662/jmaj.2022-0113

**Published:** 2022-09-12

**Authors:** Shunji Suzuki

**Affiliations:** 1 Department of Obstetrics and Gynecology, Japanese Red Cross Katsushika Maternity Hospital, Tokyo, Japan

**Keywords:** pregnancy, rubella antibody titer, rubella vaccine, Japan

Rubella is usually a mild infectious disease, often accompanied by a rash. In pregnant women, however, rubella infection can result in miscarriage, stillbirth, and a series of disabilities known as congenital rubella syndrome (CRS) ^(1), (2)^.

Rubella antibody titer <1:32, as measured by the hemagglutination inhibition (HI) test, has been considered ^(3), (4), (5)^. Therefore, in the guidelines for obstetrical practice in Japan, the administration of the rubella vaccine to nonpregnant women with a rubella HI antibody titer <1:32 has been recommended ^(6)^. However, CRS prevalence is still rising in some countries where a part of the population lacks immunity to rubella despite the presence of rubella-containing vaccines in the regular immunization program ^(7)^.

In our earlier study ^(8)^, the proportion of pregnant women with sufficient rubella HI antibody titers (≥1:32) between 2014 and 2018 was 83%-85% at our institute. Recently, many local governments in Japan, including the area where our institute is located, have provided public funding for vaccines for nonpregnant women whose rubella HI antibody titer <1:32 as preconception care ^(9), (10)^. Therefore, it is expected that the proportion of pregnant women with sufficient rubella HI antibody titers have recently increased.

Based on this background, we examined the rate of pregnant women with sufficient rubella HI antibody titers and the effectiveness of rubella vaccination as preconception care.

This retrospective study protocol was approved by the Ethics Committee of the Japanese Red Cross Katsushika Maternity Hospital (=our institute, K2019-30). Informed consent for the retrospective analysis of data was obtained from all subjects at their first perinatal visit. In our institute, all women undergo rubella HI antibody measurement with Japanese public expense during early pregnancy ^(8), (10)^. The rubella HI antibody measurement has been performed in the same lab with the same protocol in our institute since before the period of the previous study ^(8)^. At the time of the measurement, we interviewed them about their history of rubella infection and rubella vaccination.

In this study, the obstetric records of all Japanese women who underwent rubella HI antibody measurement between January and March 2021 were reviewed. We calculated the rate of pregnant women who were rubella-vaccinated with sufficient rubella HI antibody titers with or without rubella vaccination as preconception care. The data are presented as numbers and/or percentages (%).

Table 1 shows the prevalence of rubella HI antibody titers in pregnant women with and without a history of rubella vaccination as preconception care. As a whole, the proportion of pregnant women with sufficient rubella HI antibody titers was 63.4% in 2021, which decreased from that in 2014-2018 (85%-87%). Twenty pregnant women (7.5%) had been vaccinated against rubella within two years before pregnancy as preconception care; however, the rate of acquisition of sufficient rubella HI antibody titers by the vaccination was only 70% (14/20).

**Table 1.** Prevalence of rubella antibody titer using the hemagglutination inhibition test in pregnant women with and without vaccination as preconception care (at prepregnancy).

	Pregnant women with vaccination as preconception care		
	No	Yes	Total
Total Rubella antibody titer*	248	20	268
<1:8	13 (5.2)	2 (10)	15 (5.6)
1:8	32 (12.9)	1 (5)	33 (12.3)
1:16	47 (19.0)	3 (15)	50 (18.7)
(<1:32)	92 (37.1)	6 (30)	98 (36.6)
1:32	65 (26.2)	6 (30)	71 (26.5)
1:64	38 (15.3)	5 (25)	43 (16.0)
1:128	31 (12.5)	2 (10)	33 (12.3)
1:256	14 (5.6)	1 (5)	15 (5.6)
>1:256	8 (3.2)	0 (0)	8 (3.0)
(≥1:32)	156 (62.9)	14 (70)	170 (63.4)

Data are presented as numbers (percentages). *Rubella antibody titer was measured using the hemagglutination inhibition test.

Based on these results, although the awareness of prepregnancy rubella vaccination as preconception care has been gradually expanding in Japan, the effects may be incomplete.

We understand that the short study period and small sample size are serious limitations of the study. The information obtained from the interviews may not be reliable. In this retrospective study, we did not examine the detailed vaccination histories, such as routine immunization or booster vaccination. Additionally, the women who received vaccination at prepregnancy may be a population with low antibody acquisition ability against rubella.

In this study, one of the other concerns may be a subtle interpretation of the results of rubella HI tests. For example, a single dilution difference may make a significant difference in the interpretation of the results. Additionally, we cannot deny the possibility of a waning pattern of the antibody in the study subjects. Although the rubella HI test has been done with the same protocol in the same lab, we may need to keep in mind these concerns.

In this study, the current rates of sufficient rubella HI antibody titers in pregnant women following vaccination were considered insufficient. Moreover, the effect of prepregnancy rubella vaccination on women may be incomplete. To prevent and control CRS, rising awareness of vaccination campaigns regardless of sex may be necessary for Japan.

## Article Information

### Conflicts of Interest

None

### Author Contributions

Shunji Suzuki: project development, data management, data analysis, and manuscript writing/editing.

### Informed Consent

The study protocol was approved by the Ethics Committee of the Japanese Red Cross Katsushika Maternity Hospital (K2019-30).

Patients’ informed consent for the publication of this report was obtained.
